# Changes in Alpha Frequency and Power of the Electroencephalogram during Volatile-Based General Anesthesia

**DOI:** 10.3389/fnsys.2017.00036

**Published:** 2017-05-29

**Authors:** Darren Hight, Logan J. Voss, Paul S. Garcia, Jamie Sleigh

**Affiliations:** ^1^Department of Anaesthesia, Waikato Clinical Campus, University of AucklandHamilton, New Zealand; ^2^Department of Anaesthesia, Waikato District Health BoardHamilton, New Zealand; ^3^Department of Anesthesiology, Emory University School of MedicineAtlanta, GA, United States; ^4^Anesthesiology and Research Divisions, Atlanta VA Medical CenterAtlanta, GA, United States

**Keywords:** EEG, general anesthesia, alpha rhythm, frequency tuning, alpha power

## Abstract

Oscillations in the electroencephalogram (EEG) at the alpha frequency (8–12 Hz) are thought to be ubiquitous during surgical anesthesia, but the details of how this oscillation responds to ongoing changes in volatile anesthetic concentration have not been well characterized. It is not known how often alpha oscillations are absent in the clinical context, how sensitively alpha frequency and power respond to changes in anesthetic concentration, and what effect increased age has on alpha frequency. Bipolar EEG was recorded frontally from 305 patients undergoing surgery with sevoflurane or desflurane providing general anesthesia. A new method of detecting the presence of alpha oscillations based on the stability of the rate of change of the peak frequency in the alpha range was developed. Linear concentration-response curves were fitted to assess the sensitivity of alpha power and frequency measures to changing levels of anesthesia. Alpha oscillations were seen to be inexplicably absent in around 4% of patients. Maximal alpha power increased with increasing volatile anesthetic concentrations in half of the patients, and decreased in the remaining patients. Alpha frequency decreased with increasing anesthetic concentrations in near to 90% of patients. Increasing age was associated with decreased sensitivity to volatile anesthesia concentrations, and with decreased alpha frequency, which sometimes transitioned into the theta range (5–7 Hz). While peak alpha frequency shows a consistent slowing to increasing volatile concentrations, the peak power of the oscillation does not, suggesting that frequency might be more informative of depth of anesthesia than traditional power based measures during volatile-based anesthesia. The alpha oscillation becomes slower with increasing age, even when the decreased anesthetic needs of older patients were taken into account.

## Introduction

Oscillations in the human electroencephalogram (EEG) in the alpha frequency band (8–12 Hz) were first reported by Berger (1929) in awake subjects. Subsequently, alpha oscillations were also observed during sedation, sleep and anesthesia (Gibbs et al., 1937). Since these early studies, much is now known about the alpha oscillation during anesthesia. It is anteriorly located (Gugino et al., 2001), and is coherent over these areas (Cimenser et al., 2011). Alpha amplitude during anesthesia does not show strong episodic waxing and waning, as seen during stage II sleep (where it is called sleep-spindles), but rather forms a relatively sustained oscillation. Even so, the alpha oscillation of anesthesia is thought to have a similar biological origin as sleep-spindles, namely that it results from synchronized volleys of neural bursts in the thalamocortical system (Steriade et al., 1993). As the rate of neural volleys is dependent on the level of neuronal membrane hyperpolarization (Hughes and Crunelli, 2005), the frequency of an alpha oscillation is thought to represent the degree of inhibition in the thalamocortical system (Sleigh et al., 2011). The amplitude of the oscillation is likely dependent on the number of neurons recruited to act in synchrony, or the degree of synchrony itself, or both (Gibbs et al., 1937). When viewed in the frequency domain, the EEG of both sleeping and anesthetized patients is also characterized by an underlying broadband linear decrease in power (in decibels, dB) with increasing frequency, also called the spectral gradient; an oscillation will show itself as a peak of power above this broadband spectral gradient. Despite much being known about alpha during anesthesia, some key questions that have import on our understanding of how anesthetics modulate cortical networks still remain unanswered; how often does alpha occur in the clinical context, how does alpha frequency and power respond to changes in anesthetic concentration, and what is the effect of increased age on alpha frequency and power?

### Incidence of Alpha Oscillation

Alpha oscillations during anesthesia are thought to be ubiquitous (Gugino et al., 2001; Chander et al., 2014). Indeed, alpha oscillations reliably show up in the anesthetized EEG during controlled experimental studies with propofol (Ní Mhuircheartaigh et al., 2013; Purdon et al., 2013), and replication of the alpha oscillation remains the main goal of computational models seeking to understand the mechanisms of anesthesia (e.g., Ching et al., 2010; Liley et al., 2011). Nonetheless, an often overlooked clinical observation is that the frontal EEG of some anesthetized patients under a volatile-based anesthetic do not display any discernible peaks of power in the alpha range at all. It is currently unclear what proportion of patients do not show alpha activity in the clinical situation.

### Responsiveness of Alpha Power Measures to Anesthetic Concentration

One of the most common measures of the alpha signal is the magnitude of power over the alpha range (usually 8–12 Hz). The problem with this simple measurement is that it will conflate both the underlying broad-band noisy activity (1/*f*), as well as any specific narrow-band oscillations. It is likely that these patterns reflect at least two different underlying neurobiological processes. Leslie et al. (2009) looked specifically at the oscillatory component of the alpha oscillation, i.e., the power of the alpha oscillation *additional* to the underlying broadband spectral gradient. While it is known that the steepness of the spectral gradient itself changes with anesthetic concentration (Jospin et al., 2007), there is to our knowledge, no in-depth analysis of how the magnitude of the alpha oscillation responds to changes in volatile anesthetic concentration during surgical anesthesia, nor its relationship to changes in the underlying spectral gradient.

### Concentration-Responsiveness of Alpha Frequency and the Effect of Age

During the transition into and out of general anesthesia, the frequency of the alpha oscillation shifts according to anaesthetic concentration, as observed by Long et al. (1989) and more recently by Purdon et al. (2013). The latter work shows that with propofol, the spectral median, a close approximation to peak frequency, reaches a lower frequency limit during deeper stages of anesthesia. Clinical observations suggest that during a volatile based anesthetic, at least in some patients, the frequency of the alpha oscillation remains sensitive to volatile anesthetic concentration well into surgical doses. While age has been shown to decrease alpha frequency for a given anesthetic concentration (Purdon et al., 2015a), the precise effect of age on the shifting alpha frequency in response to a changing anesthetic concentration during surgery remains unknown.

The aims of this study are therefore threefold:

1. To note the incidence of alpha oscillatory activity in a clinical population receiving a volatile anesthetic.
2. To characterize changes in the oscillatory components of alpha power (peak, broadband and oscillatory alpha power), alpha frequency, and the spectral gradient to changing volatile anesthetic concentration.
3. To characterize the effect of age on the relationship between alpha frequency and volatile anesthetic concentration.

## Materials and Methods

This observational study was approved by the New Zealand Health and Disability Ethics Committee (Ref. 12/CEN/56), and all patients gave informed written consent before being included. Physician anesthetists were allowed to provide anesthetic care according to their own clinical judgment. Volatile gas anesthetic (VGA) concentrations at the end of each breath cycle (end-tidal) were recorded every 5 s from the S5 Anesthesia Monitor using the S5 Collect program (both from GE Healthcare, Helsinki, Finland) in units of Minimum Alveoli Concentration (MAC) to allow comparison between different gas types. Brain effect-site VGA concentrations were estimated using a delay model with a half-time equilibrium constant (*K*_eo_) of 144 s as reported in McKay et al. (2006), and named C_e_MAC. VGA concentrations were adjusted for age according to norms set out in Nickalls and Mapelson (2003). The timing and doses of opioid analgesics (fentanyl or morphine) were noted, and effect-site opioid concentration estimated using two-compartment models (Shafer and Varvel, 1991, for fentanyl and Mazoit et al., 2007, for morphine), with 2 mg of morphine seen as equivalent to 100 μg fentanyl.

### EEG Recordings

Frontal bipolar EEG (with FP_Z_ as reference, and either FP_1_ or FP_2_ as active electrode) was recorded from 305 patients undergoing surgery at the Waikato District Health Board in Hamilton, New Zealand using either a Bispectral Index^®^ (BIS^®^, from Aspect Medical Systems, Newton, MA, USA, with a sampling rate of 128/s) or Entropy (GE Healthcare, Helsinki, Finland, sampling rate: 100/s) anesthetic depth monitor. Researchers interested in analyzing data from this database can submit requests online at www.accesshq.org. A single straight-line fit over the entire EEG was then subtracted from the raw EEG (i.e., detrended) and then filtered with high (0.25 Hz) and low (48.5 Hz) pass 3rd order Butterworth filters (using the phase-preserving filtfilt.m function) in MATLAB (Version R2016a, The MathWorks, Inc., Natick, MA, USA). BIS recordings were then down-sampled to 100 Hz to allow comparison with Entropy recordings. The presence of burst suppression was detected, where periods of the EEG with amplitudes less than 5 μV for more than 0.5 s were considered supressed.

### Spectral Analyses

A Fast-Fourier Transform (FFT) was used to calculate power spectral densities, using the multi-taper method developed by Thomson (1982), and available in the Chronux toolbox for MATLAB (Bokil et al., 2010). We chose a moving window of 10 s length, with an offset of 1 s. A 0.25 Hz frequency resolution allowed the reasonable use of four tapers. Burst suppression was considered as any period of the EEG where amplitudes remained below 5 μV for more than 0.5 s.

**Figure 1:**
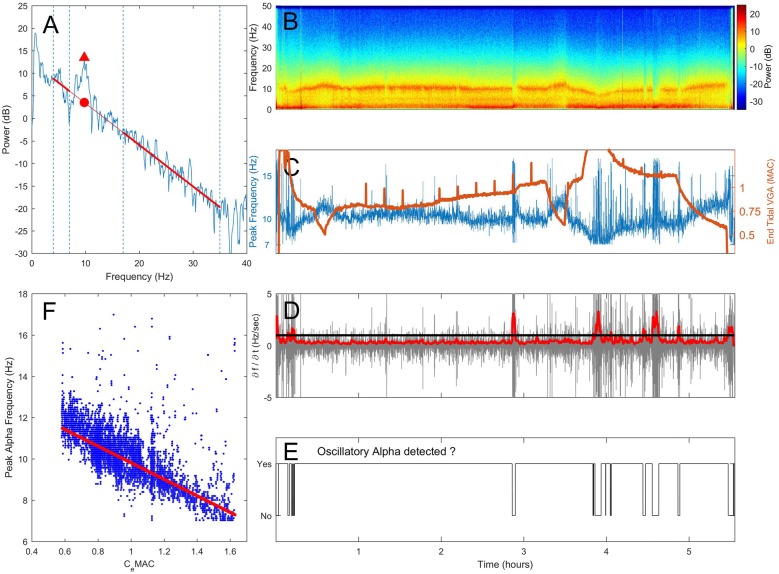
**Proposed method of assessing the presence or absence of alpha oscillations. (A)** A section of electroencephalogram (EEG) is transformed into the frequency domain using a Fast-Fourier Transform (FFT). Spectral gradient (red line) is fitted via linear regression to the spectrum omitting the delta (0.5–4 Hz) and extended alpha (7–17 Hz) where oscillations are often present. The frequency and power at the maximal power above the spectral gradient (triangle) are recorded. An example spectrogram from one patient over a 5.5 h operation is shown in **(B)**. In **(C)** changes in peak frequency (blue, left axis) and anesthetic concentration (end-tidal minimum alveoli concentration (MAC), orange, right axis) are shown against time. The rate of change of peak frequency (**D**, gray) was median smoothed (**D**, red). When the smoothed first derivative of frequency breached a threshold of 1 standard deviation, oscillatory alpha was classified as absent **(E)**. The concentration-response relationship was determined by fitting a robust regression to peak alpha frequency against C_e_MAC **(F)**, but only for when an alpha oscillation was classified as present in **(E)**.

As the precise relationship between narrowband alpha power and the underlying broadband spectral gradient during changing levels of anesthesia is not known, we used the convention set out in Leslie et al. (2009). Here, the slope of the spectral gradient is computed by fitting a linear regression line to the power over the subset of frequencies not influenced by the delta and alpha oscillations (i.e., to power within the 4–7 Hz, and 17–35 Hz frequency range, see Figure 1A). The frequency at which the alpha power above the underlying broadband power is greatest within this extended alpha range (7–17 Hz), is the peak frequency. At this frequency, the peak alpha power is the power at the peak of the oscillation (Figure 1A, triangle), and broadband alpha power refers to the power on the broadband spectral gradient also at that frequency (Figure 1A, circle); oscillatory alpha power is the difference between the peak and broadband alpha power (i.e., the size of the oscillation above the broadband spectral gradient).

### Detecting Oscillatory Alpha Activity

When a genuine sustained oscillation in the alpha range is present, peak alpha power will be consistently higher than the spectral gradient, and peak frequency will be centered on a stable frequency. If the peak alpha frequency suddenly shifts, this indicates that peak frequency has jumped from one oscillatory peak to another within the 7–17 Hz allowable range, and that the oscillatory alpha power is within the range of noise on the spectral gradient. In this way, a maximal value of the rate of change in peak frequency can be used as a threshold to assess if a sustained oscillation is present or not. Note that if the peak frequency of the oscillation is not stable at a given frequency, a value of oscillatory alpha power will still be given. In this instance the oscillatory alpha power value will be a measure of the noise in the spectrum around the spectral gradient and thus depend on the time window length and frequency resolution chosen.

Practically,

1. Peak alpha frequency was measured from the spectrum (Figure 1A, spectrogram in 1B) and displayed over time (Figure 1C, blue line).
2. The first derivative of the peak alpha frequency (the rate of change of peak frequency per second) was calculated (Figure 1D, gray line). The standard deviation of the first derivative in 10 s moving widows (offset of 1 s) was used to give an absolute measure of spread of frequency change rates, which was then median filtered (order of 80 s) to smooth out any sudden but transient changes (Figure 1D, red line).
3. When a threshold of 1 standard deviation of change in peak frequency was breached, sustained oscillatory alpha activity was considered absent (Figure 1E).
4. Individual concentration-response slopes were calculated by fitting a linear robust regression (using a bisquare weighting function with a tuning constant of 4.685) of peak alpha frequency against C_e_MAC (Figure 1F, C_e_MAC also shown in 1C, orange line) for where oscillatory alpha was detected. We note that the linear fit we have employed is a first approximation of the data. As our data was observational, and recorded from the unconstrained clinical environment, other pharmacological or surgical interventions can lead to non-stationarities in the frequency-concentration relationship.
5. Concentration-response relationships were also fitted for peak (Figure 1A, triangle) and broadband (Figure 1A, circle) alpha power. The difference in power was the oscillatory alpha power.

Our analysis began at start of surgery and ended at the beginning of gas flush, and any period of EEG showing burst suppression did not enter the concentration-response regression. All correlations were calculated using Pearson’s Correlation Coefficient.

## Results

Of the original 305 patients, 19 patients were excluded as dropouts, 18 rejected due to problems with VGA recordings, and 10 excluded as they received a non-volatile based anesthesia (propofol).

Of the 258 patients included for analysis, patient ages ranged from 18 years to 90 years (median 64 years, interquartile range 27 years). Patient gender was split evenly (128 females, 130 males), and the majority of patients received sevoflurane (188, 73%) with the remaining receiving desflurane. A wide range of surgical disciplines entered the analysis, the most prevalent being general surgery (41%), followed by vascular surgery cases (26%), with gynecology and urology (25%). The remaining surgery types (8%) were plastics, thoracic, with one ENT and neurological case each. Median operation length was 71 min (IQR 104 mins), and the median C_e_MAC over the operation for all patients was 0.96 (IQR 0.22). Median effect-site opioid concentration during the operation was 0.81 ng/ml fentanyl-equivalents (IQR 0.74).

### Incidence of Alpha in a Clinical Population with Volatile Anesthetics

An ongoing oscillation in the alpha range was not always present; 48 patients (19%) showed alpha activity for less than half of the surgical anesthesia period. Retrospective *post hoc* inspection of their spectrograms revealed that 18 of these patients (36% of this subgroup) had either burst-suppression (7 patients) or a delta and theta dominant EEG (here classified as between 4 Hz and 6 Hz, 11 patients), or both. Seventeen patients (35%) had a weak alpha oscillation visually evident in the spectrogram that our algorithm could not detect, and 3 (6%) a noisy recording, likely from muscle activity or electrocautery. Only 10 patients (21% of this subgroup, or 4% of all patients accepted into the analysis) had an absence of oscillatory alpha power that could not be linked to the aforementioned causes. The median age of these 10 patients (74 years) was older than that of all remaining patients (63 years, *p* = 0.03). These patients did not have higher median C_e_MAC concentrations during the operation (0.99 vs. 0.95, *p* = 0.52), even when C_e_MAC was age-adjusted (1.18 vs. 1.07 C_e_MAC, *p* = 0.15). Median effect-site opioid concentration was not lower in this patient group compared to the remaining patients (both groups 0.81 ng/ml, *p* = 0.77, all comparisons using the Mann-Whitney-*U test*).

### Limits of the Alpha Oscillation

In our analysis, concentration-response curves were only fitted to C_e_MAC values when an alpha oscillation was detected. Patients may have received higher C_e_MAC concentrations over the operation than the concentrations where alpha was observed. In this instance (where the maximal concentration over the operation was higher than the maximal concentration where alpha was observed) the maximal C_e_MAC value of the concentration-response fitting represents the limit at which alpha oscillations occur for that patient. This is in contrast to where alpha oscillations were observed right up until the highest C_e_MAC value that patient received over the operation; in this instance the maximal C_e_MAC could simply represent the maximal concentration the anesthetist was willing to give to that patient, and not the natural concentration limit of the alpha oscillation *per se*. In our data, a subset of 21 patients received at least 0.1 C_e_MAC more than the maximal C_e_MAC of the concentration-response regression. In this patient subgroup (median age 73 years, IQR 20 years) the median maximal C_e_MAC values of the concentration-response regression was 1.01, with an inter-quartile range of 0.29 C_e_MAC. The mean maximal age-adjusted C_e_MAC value was 1.17 (IQR 0.30) and as such represents an estimate of the upper volatile anesthetic concentration limits where the alpha oscillation occurs.

### Concentration-Response Results

The following results focus on the sign of the concentration-response slope, where a positive concentration-response slope indicates an increase in frequency or power, and a steepening of the spectral gradient to increasing anesthetic concentration, a negative concentration-response slope a decrease in the same measures to increasing anesthetic concentration. For the concentration-response analysis the 48 patients with alpha activity for less than 50% of the maintenance anesthesia period were excluded. A further 46 patients were also rejected due to having only a very small C_e_MAC range (0.15 C_e_MAC during surgery), which made fitting the linear regression unreliable. This *post hoc* criteria was necessary as we observed (unpublished data) that many patients with a C_e_MAC range less than this value had non-physiological concentration-response slope values. C_e_MAC values are non-age-adjusted unless otherwise mentioned. There was no statistically significant effect of type of volatile anesthetic on the concentration-effect slopes for alpha frequency, any alpha power measures, or spectral gradient (all *p-values >0.05*).

### Concentration-Response Relationship for Peak Alpha Frequency

**Figure 2:**
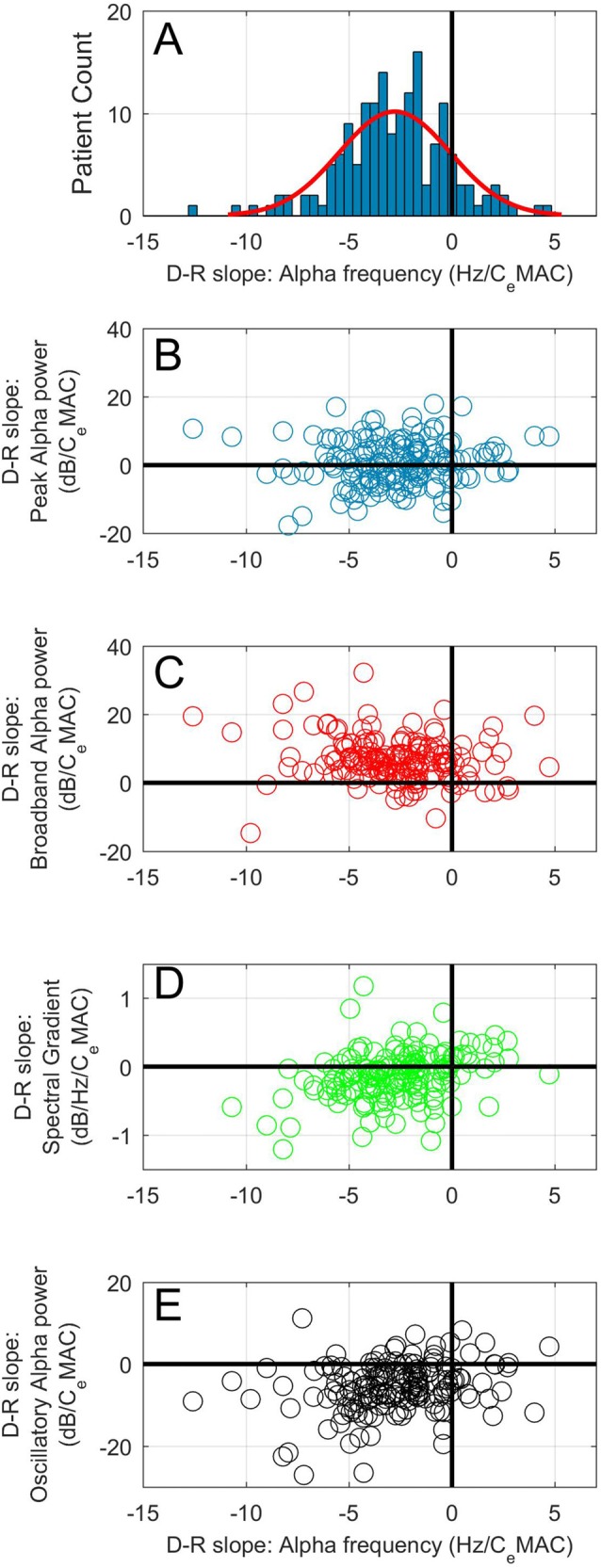
**(A)** Histogram of concentration-response slopes for alpha frequency to C_e_MAC. Scatterplots of concentration-response slopes for peak alpha frequency against: peak alpha power **(B)**, broadband alpha power **(C)**, spectral gradient **(D)** and oscillatory alpha power **(E)**.

The mean patient C_e_MAC where an alpha oscillation was present was 0.84, and the mean peak alpha frequency at this C_e_MAC concentration was 9.2 Hz. The mean concentration-response slope for alpha frequency was −2.8 Hz/C_e_MAC (SD 2.7), indicating a near to 0.5 Hz frequency slowing with each 0.2 C_e_MAC increase in anesthetic concentration. Eighty-eight percent of patients (145/164) had a negative concentration-response slope for alpha frequency. Figure 2A shows the distribution of the concentration-response slopes for peak alpha frequency. Spectrograms with peak alpha frequency and anesthetic concentrations for four additional example patients are shown in Supplementary Figure S1. Median power spectra from six example patients taken from a 1-min period just following cessation of surgery can be seen in Supplementary Figure S2. The effects of age on alpha frequency are shown in a later section.

### Concentration-Response Relationship of Peak Alpha Power

The mean peak alpha power at mean C_e_MAC (0.84 MAC) was 12.4 dB. An even distribution of positive and negative concentration-response slopes for peak alpha power were noted to increasing anesthetic concentration (Figure 2B). The mean concentration-response slope for peak alpha power was 0.6 dB/C_e_MAC (SD 6.8). Eighty-six (52%) patients increased peak alpha power with increasing anesthetic concentration, whilst 78 (48%) showed the opposite—a decrease. Magnitude of the peak alpha power-C_e_MAC concentration-response slope was not associated with age, maximal or minimal C_e_MAC, maximal or minimal peak alpha frequency, mean opioid concentration (all *p* values >0.15 using the Pearson’s correlation coefficient test), and neither with gender, or gas-type (both *p*-values >0.5, Mann Whitney-*U* test). A statistically significant correlation between magnitude of concentration-response slope and length of operation was noted (*R* = 0.19, *p* = 0.015) but this association could only explain 3.6% of the variance in slope magnitude.

### Concentration-Response Slopes of Broadband Alpha Power and Spectral Gradient

The mean broadband alpha power at mean C_e_MAC was 3.3 dB. The mean concentration-response slope for broadband alpha power was 6.8 dB/C_e_MAC (SD 6.4). One-hundred and fourty-four patients (88%) had a positive concentration-response slope for broadband power (see Figure 2C). Concentration-response slope for broadband power was not correlated with age (*p* = 0.61).

Given that the majority of patients (also 88%) showed a decrease in alpha frequency to increasing C_e_MAC, we would expect broadband alpha power to increase to increasing C_e_MAC simply due to the point of measurement sliding up the negative spectral gradient (see Figure 1A, where a slowing in peak frequency (▲) necessarily leads to an increase in broadband power (●)). To add complexity to the situation, the steepness of the spectral gradient itself was also concentration-dependent (Figure 2D). The mean spectral gradient at mean C_e_MAC was −0.92 dB/Hz, and the mean concentration-response slope for spectral gradient was −0.19 dB/Hz/C_e_MAC (SD 0.61). Two-thirds of patients (110/164) had negative concentration-response slopes, indicating an increasing steepness of the spectral gradient to increasing C_e_MAC, and one third (54/164) had positive slopes. The concentration-response slope of the spectral gradient was not correlated with age (*p* = 0.10).

### Concentration-Response Slopes of Oscillatory Alpha Power

The mean oscillatory alpha power at mean C_e_MAC was 8.9 dB. The mean concentration-response slope for oscillatory alpha power was −5.7 dB/C_e_MAC (SD 6.1). One-hundred and fourty-one patients (86%) had a negative concentration-response slope for oscillatory alpha power (Figure 2E).

### The Effect of Age on the Concentration-Response Relationship for Peak Alpha Frequency

**Figure 3:**
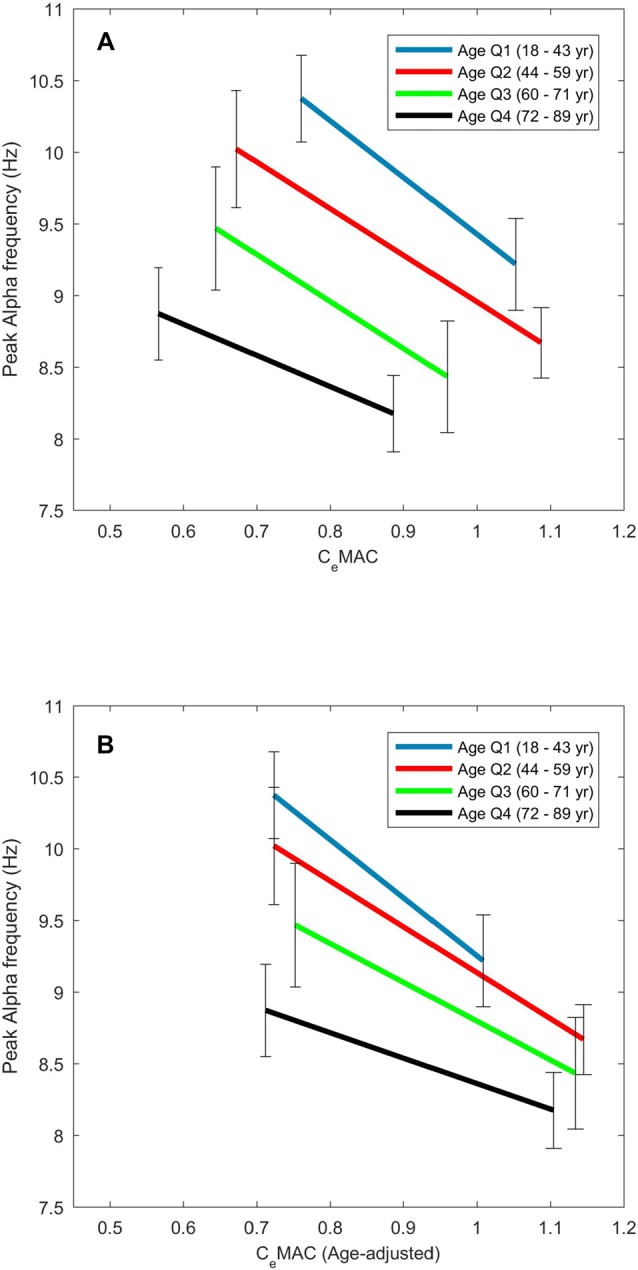
**The effect of age on the concentration–response slopes for alpha frequency.** Median values are displayed for each age quartile for C_e_MAC **(A)** and age-adjusted C_e_MAC **(B)**.

The maximum and minimum peak alpha frequency of the concentration-response curves both decreased with age (*R* = −0.47 and −0.46 respectively, *p*-values both <0.001). The maximum and minimum C_e_MAC values of the concentration response curves also decreased with age (*R* = −0.37 and −0.41 respectively, *p*-values both <0.001). The slope of the concentration-response curve became less negative with increasing age (*R* = 0.17, *p* = 0.030). This effect of increasing age on the concentration-response measures is shown in Figure 3A, where age has been divided into quartiles. Between the youngest (18–44 years) and oldest (72–89 years) age quartiles the median maximum alpha frequency decreased from 10.4 Hz to 8.9 Hz, and the median concentration-response slope decreased from −3.5 Hz/MAC to −1.9 Hz/MAC. The decreasing peak frequencies and C_e_MAC concentrations with age result in a left shift of the concentration-response curves.

When age-adjusted C_e_MAC values were used, minimum C_e_MAC did not change with age (*R* = 0.05, *p* = 0.57), but the maximum C_e_MAC values of the concentration response curves now increased with age (*R* = 0.26, *p* < 0.001). Concentration-response slopes continued to become less negative with age, though the association was stronger (*R* = 0.29, *p* < 0.001) with age-adjusted values. The left-shift of the concentration-response curves to increasing age (Figure 3A) is thus transformed into a downward-shift (Figure 3B) when age-adjusted C_e_MAC values are used.

In summary, in most patients, peak alpha frequency decreases in response to increasing anesthetic concentration. While peak alpha power can either increase or decrease with increasing anesthetic concentration, broadband alpha will usually increase, resulting in a decreasing oscillatory alpha power. The increase in broadband alpha power is not simply a mathematical consequence of a slide up the negative spectral gradient with slowing alpha frequency, but is contributed to in some degree by an increasing steepness of the spectral gradient itself with increasing anesthetic concentration.

## Discussion

This study reveals new findings concerning the incidence, concentration-responsiveness, and effect of age on frontal oscillatory alpha activity during volatile based clinical anesthesia.

A frontal alpha oscillation under anesthesia was inexplicably absent in around 4% of our patients. This rate is slightly lower than some older studies looking at occipital alpha in awake patients with eyes closed (summarized in Vogel and Götze, 1959), where absence ranged between 7% and 11%), but does point toward a genetic or neural-degeneration explanation, rather than an anesthetic dosing effect. These patients were older, but did not have a deeper anesthetic, nor a higher opioid dose. In our analysis, we did not consider patients with a delta and theta dominant EEG as having an unexplainably absent alpha oscillation, as we assumed that this EEG state only occurs during quite deep anesthesia close to burst-suppression based on clinical observations. If this assumption was proved incorrect, the percentage of patients with unexplainably absent alpha would increase to 8%. We also note that in the clinical situation insufficient analgesia could lead to an absence of alpha activity (as seen in Hagihira et al., 2004; MacKay et al., 2010; Sleigh et al., 2010), although this effect is generally transient; we could find no good evidence of inadequate opioid levels in our data.

That alpha power only increases to increasing anesthetic concentration in half our patients is a novel finding. Most previous studies have focussed on the induction or emergence period where large changes in anesthetic concentration and behavioral response are guaranteed (e.g., Purdon et al., 2013; Chander et al., 2014). In some studies where anesthetic concentrations were increased to surgical levels (Gugino et al., 2001; Kuizenga et al., 2001), an initial increase and subsequent decrease in alpha power to increasing anesthesia concentrations has been noted—a biphasic response. This effect has also been replicated in some model-based studies (Hutt, 2011). In these cases, a linear fit such as we chose would not be appropriate for a non-monotonic response, and the slope of the concentration-response fit might depend on anesthetic concentration, i.e., positive concentration-response slopes might be a response to lighter levels of anesthesia, and negative concentration-response slopes to deeper. We did not observe this in our dataset; both the maximal and minimal values of the C_e_MAC range where alpha was observed were not correlated with sign of the concentration-response curve slope, and discounts this biphasic effect as an explanation. Inspection of the concentration response figures showed that the more traditional use of a sigmoid fitting was not warranted for the alpha power measure; in a few cases (around 8 patients), alpha power saturated at a maximal level, and the use of a sigmoid would have been more suitable, but the sign of the concentration-response slope would not have changed, and is very unlikely to change the results.

That alpha power increases for only half of our patients may also be due to a limitation of spectral analysis, such as the effect of a non-sinusoidal shaped waveform. An increase in peak spectral power could be caused by an alpha waveform increasing in amplitude, or may also be due to an alpha waveform with the same amplitude becoming more tightly sinusoidal. Alternative methods, such as wavelets, would be needed to tease out these subtle effects of wave morphology.

In contrast to alpha power, the slowing of alpha frequency to increasing anesthetic concentration in the majority of patients is a clear finding, and suggests that frequency might be more informative of depth of anesthesia than traditional power measures during volatile-based surgical anesthesia.

Slowing of the occipital alpha frequency can also be seen in response to subtle physiological changes such as decreased temperature (e.g., Chang et al., 2011), and is also associated with various clinical conditions such as Parkinson’s, Alzheimer’s and depression (Niedermeyer, 1997; Llinás et al., 1999); importantly, in these cases, the alpha frequency can slide into the theta range (classically 5–7 Hz). The frontal alpha slowing under anesthesia in our study was linearly related to anesthetic concentration, and slid seamlessly into the theta range. Inspection of the concentration response figures showed that the more traditional use of a sigmoid fit was not warranted for alpha frequency; alpha frequency does not seem to reach a lower frequency bound under volatile based anesthesia, as it does with propofol (Purdon et al., 2013).

Regarding the possible mechanisms of alpha slowing, Hughes and Crunelli (2005) demonstrated that for occipital alpha, the frequency manifest in the EEG was dependant on the degree of hyperpolarization in thalamocortical cells. In slices, the oscillation frequency ranged seamlessly from 2 Hz to 13 Hz as a result of changing levels of depolarization. Some recent modeling studies have also focussed on shifting alpha frequency. The shifting peak frequency of an oscillation has been shown to be responsive of the level of stimulation entering into a neural network (Cohen, 2014), and is also sensitive to the nature of the input to a neuronal circuit (Lefebvre et al., 2015), but could also be due to a prolongation of the inhibitory post-synaptic potential.

A decrease in alpha frequency under sevoflurane anesthesia with increasing age has been previously noted by Purdon et al. (2015a) who observed a 0.5 Hz slowing between the group means of young (18–38 years) and elderly (70–90 years) patients who all received approximately 1 MAC when age-adjusted. Hindriks and van Putten (2012) noted that when giving a propofol anesthetic for cardiac surgery (which involves mostly older patients) peak alpha frequency was slower than the classic 8–12 Hz band, and chose 6 Hz as the lower frequency bound for alpha power. In our study, we have shown that not only do the frequency bounds of the alpha oscillation decrease with age, the sensitivity of the frequency change to anesthetic concentration also decreases with age, i.e., the concentration-response slopes becomes less steep with increasing age (see Figure 3). To some measure this decreased slope could be due to our arbitrary 7 Hz lower limit for the alpha frequency. Additionally, if the alpha oscillation does shift into the classical theta range (4–7 Hz) it will increase the spectral gradient measure; this stands as a limitation to our chosen method. We suspect that this effect only occurs in a small subset of patients, but this has not been quantified. That the left-shift of the concentration-response slopes with increasing age becomes a downward-shift when age-adjusted MAC values are used implies a reasonable agreement between the original clinical measure of MAC (the presence of movement to a two centimetre skin incision) and our alpha frequency based cortex measure.

The spectral gradient itself was sensitive to anesthetic concentration in two-thirds of our patients. We chose to use a log-linear fit for the spectral gradient as this historically had a better fit to the data than the log-log fit (Sleigh et al., 2010). Also, this method has two further advantages; first, that power in the slower frequencies did not overly dominate the regression as is possible in a log-log regression, and second our sampling rate of 100 per second necessarily limited the range of frequencies available for a fit. Although there are different methods available for normalizing the spectral gradient to find the oscillation, such as finding the difference between electrodes (Werth et al., 1997), or using a differentiator filter (Demanuele et al., 2007), the main contribution of this study is to emphasize that the spectral gradient itself can be responsive to anesthetic concentration. With any method of spectral normalization, if it is being applied over changing anesthetic concentrations, this observation would need to be taken into account.

A number of observations in this study also have relevance to any depth of anesthesia measures based on the alpha waveform. While the indexes of the two current primary commercial monitors (the Bispectral Index, or BIS^®^, and Entropy Module from GE Healthcare) are not based specifically on the alpha waveform, some newer proposals are (for example Sleigh et al., 2011; Mukamel et al., 2014). During a volatile based anesthetic we can expect around 4%–8% of patients to not show a frontal alpha oscillation. Also, in older patients, the alpha frequency can slide into the classic theta range (i.e., below 7 Hz), and alpha-based measures would need to accommodate this frequency slippage. Any measures dependent on bandpass filtering will have to be broad enough to “follow” the frequency of the oscillation, but may suffer from signal to noise issues as a result. In contrast, a spectrogram is particularly suited to observing frequency shifts in oscillations (see for example Figure 1B), and in the clinical situation can give an anesthetist an immediate feel for the state of the cortex of their patient (Purdon et al., 2015b).

To conclude, in this study we have demonstrated that clear oscillatory alpha activity was absent in near to 5% of our clinical population, and that while peak alpha frequency shows a consistent slowing to increasing volatile gas concentration during surgical anesthesia, the peak power of the oscillation does not, only increasing in around half of the patient group. The underlying broadband spectral gradient became steeper with increasing concentration in two-thirds of patients. We have also shown that the alpha oscillation becomes slower with increasing age, even when the decreased anesthetic needs of older patients were taken into account.

## Author Contributions

DH completed EEG recordings and analysis and wrote the manuscript. LJV, PSG and JS wrote the manuscript.

## Conflict of Interest Statement

The authors declare that the research was conducted in the absence of any commercial or financial relationships that could be construed as a potential conflict of interest.

## Supplementary Material

FIGURE S1 **Example data from four additional patients (A–D)** showing spectrograms and the relation between anesthetic concentration and peak alpha frequency. Note that for ease of viewing anesthetic concentration has been inverted on the *y*-axis. Patients **(A–C)** show clear relationships between anesthetic concentration and frequency, but patient **(D)** does not.

FIGURE S2 **Power spectra for six example patients (A–F)** showing median power (thick blue line) from a 60 s period following cessation of surgery.
