# Supplementary figures and images for: Changes in Alpha Frequency and Power of the Electroencephalogram during Volatile-Based General Anesthesia
Source: Front Syst Neurosci. 2017 May 29;11:36. doi: 10.3389/fnsys.2017.00036 (PMC5446988; doi:10.3389/fnsys.2017.00036)

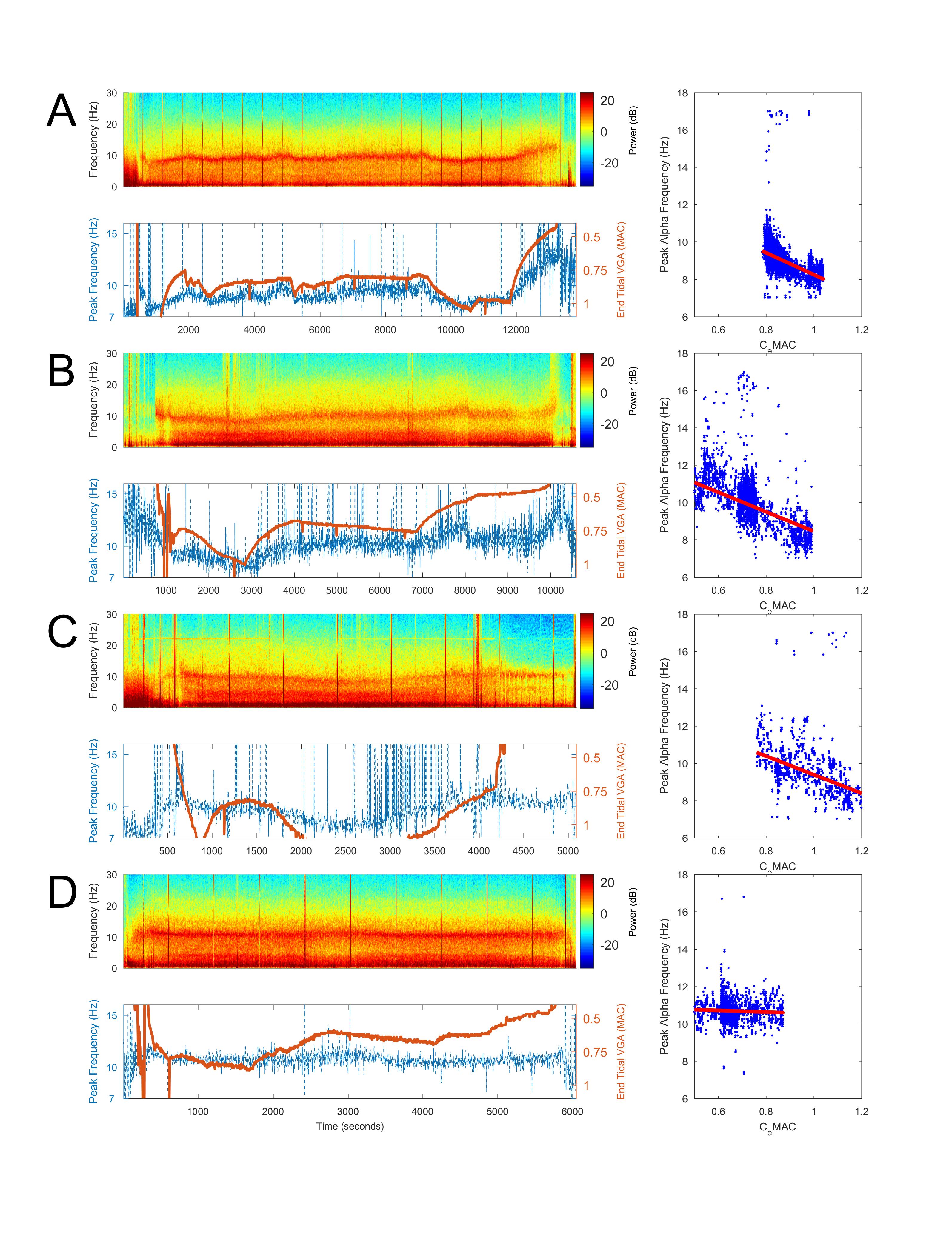

Supplement: FIGURE S1 — Example data from four additional patients (A–D) showing spectrograms and the relation between anesthetic concentration and peak alpha frequency. Note that for ease of viewing anesthetic concentration has been inverted on the y-axis. Patients (A–C) show clear relationships between anesthetic concentration and frequency, but patient (D) does not. [file Image_1.jpeg]

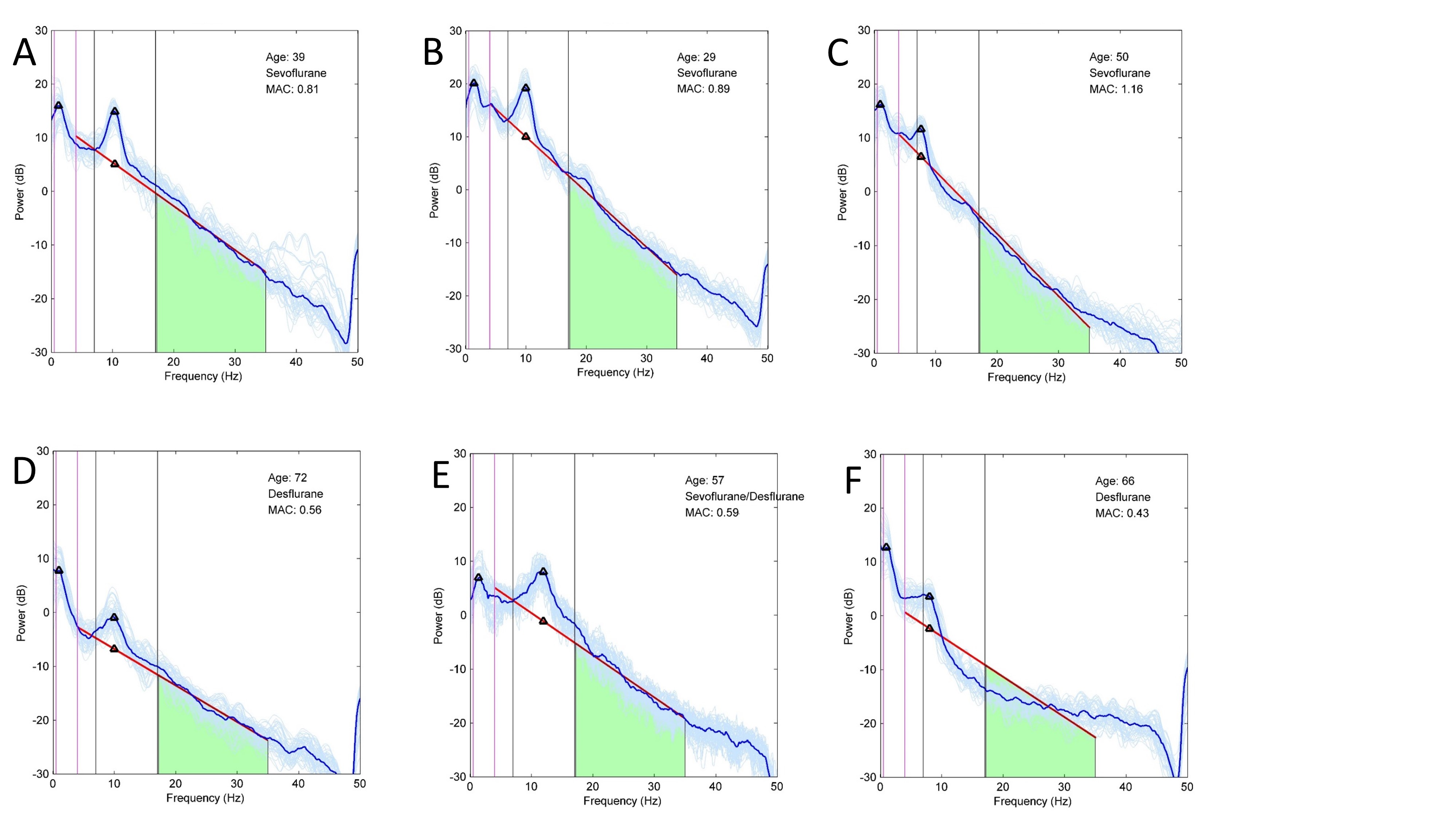

Supplement: FIGURE S2 — Power spectra for six example patients (A–F) showing median power (thick blue line) from a 60 s period following cessation of surgery. [file Image_2.jpeg]
